# Fertility-sparing strategy in a rare case of highly malignant Dicer-1-associated sarcoma of the cervix

**DOI:** 10.1007/s00404-024-07588-x

**Published:** 2024-07-08

**Authors:** J. Altmann, K. Kubiak, J. Sehouli, E. Roser

**Affiliations:** 1 Department of Gynecology with Center of Oncological Surgery, Charité-University Hospital Berlin, Charitéplatz 1, 10117 Berlin, Germany; 2 Department of Gynecology, St-Franziskus Hospital, Münster, Germany

**Keywords:** Dicer-1 sarcoma, Cervical sarcoma, Fertility sparing, Fertility preservation

## Abstract

**Introduction:**

We present the rare case of an 18-year-old patient with a Dicer-1 mutation-associated sarcoma of the cervix uteri.

**Case:**

The patient presented with irregular vaginal bleeding in July 2022. The clinical examination showed an exophytic tumor of the cervix, uterus and ovaries were normal in sonogram. The tumor of the cervix was resected, followed by a diagnostic hysteroscopy and abrasion of the uterine cervix and cavity. Hysteroscopy showed normal findings of the cervix and uterus. After diagnosis of a highly malignant Dicer-1 mutation-associated sarcoma of the cervix, cryopreservation of oocytes was realized. Based on the principle of obtaining maximum oncological safety while preserving fertility in this 18-year-old patient, we recommended chemotherapy rather than radiation with its far severe implications on the patient´s reproductive organs. 4 cycles of chemotherapy consisting of doxorubicin and ifosfamide were applied until December 2022. After re-staging in December 2022 via CT scan and MRI, the abdomen and pelvis as well as control hysteroscopy and abrasion were unremarkable. Until now, the patient is tumor free.

**Discussion:**

Primary sarcomas of the cervix are very rare. Recent literature hints towards a distinct DICER-1 sarcoma entity characterized by specific mutational clusters. Limited follow-up data suggested that DICER1-mutant tumors might exhibit a less aggressive clinical course than DICER1-wild-type tumors.

**Conclusion:**

Decision-making in case of rare histological entities with sparse recommendations in the literature poses a challenge to the treating physician. Treatment strategies should consider oncological safety as well as options of preserving fertility. Gonadotoxic potential of different strategies should be taken into consideration and discussed in detail with the affected patient.

## What does this study add to the clinical work

**Table:** 

Decision-making in case of rare histological entities with sparce recommendations in the literature poses a challenge to the treating physician. Treatment strategies should consider oncological safety as well as options of preserving fertility.	

## Introduction

Primary sarcomas of the cervix account for approximately 1.3% of cervical tumors [1]. The vast majority of histologic subtypes of sarcomas of the cervix are carcinosarcoma (50%) followed by leiomyosarcoma and adenosarcoma, the remaining ~ 9% are a heterogenous group of sarcomas with survival rates being significantly worse for the last group [1].

We report the case of a Dicer-1 mutation-associated sarcoma of the cervix and discuss treatment options with emphasis on fertility preservation in this young female patient.

## Case report

**Fig. 1:**
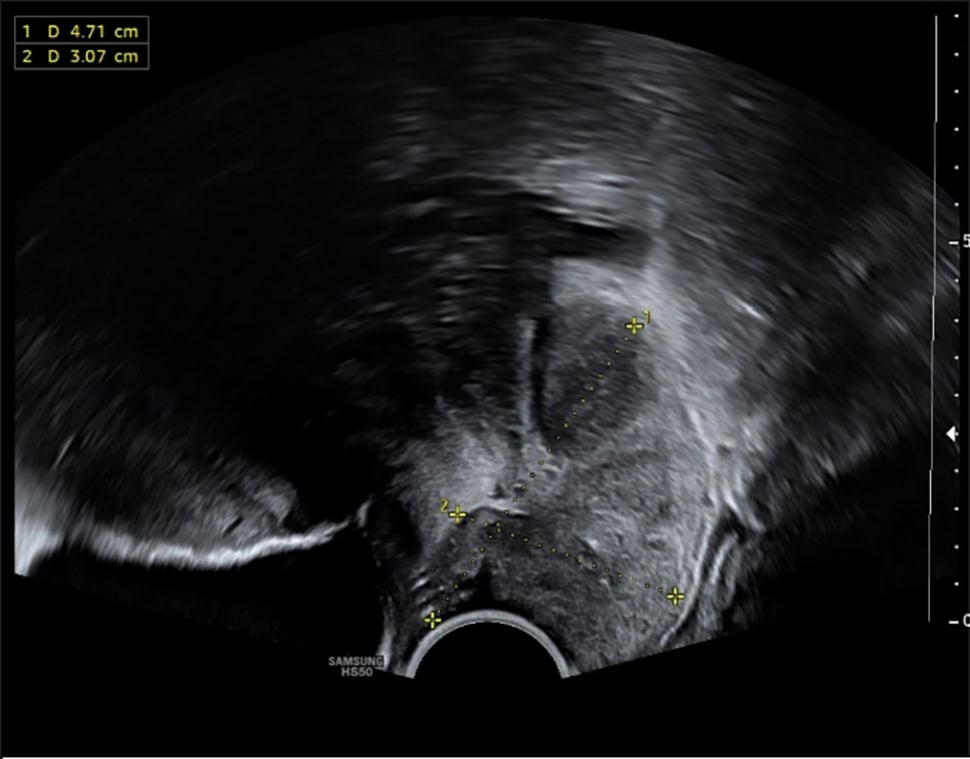
Transvaginal sonogram shows an exophytic tumor of the uterine cervix

**Fig. 2:**
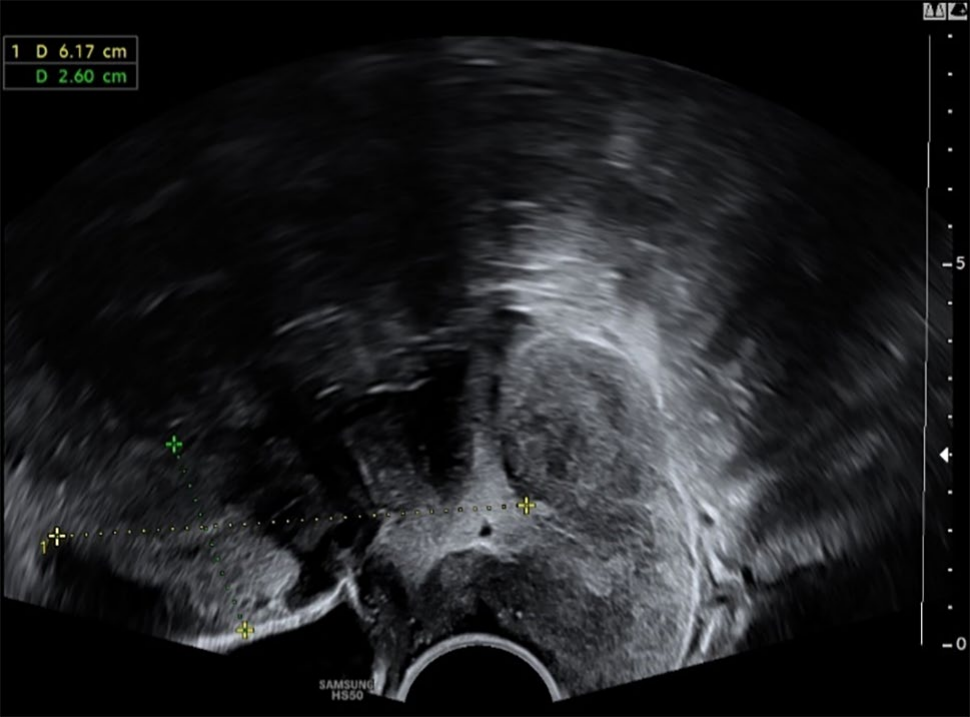
Transvaginal sonogram of the uterus and tumor of the cervix

**Fig. 3:**
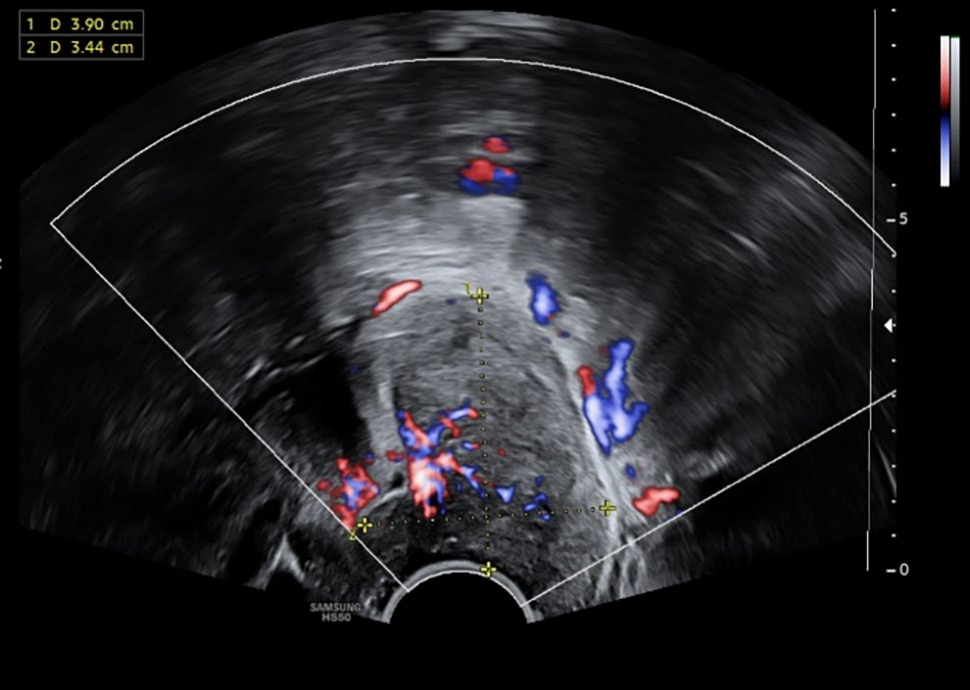
Transvaginal duplex sonography shows intensive vascularization of the tumor of the uterine cervix

**Fig. 4:**
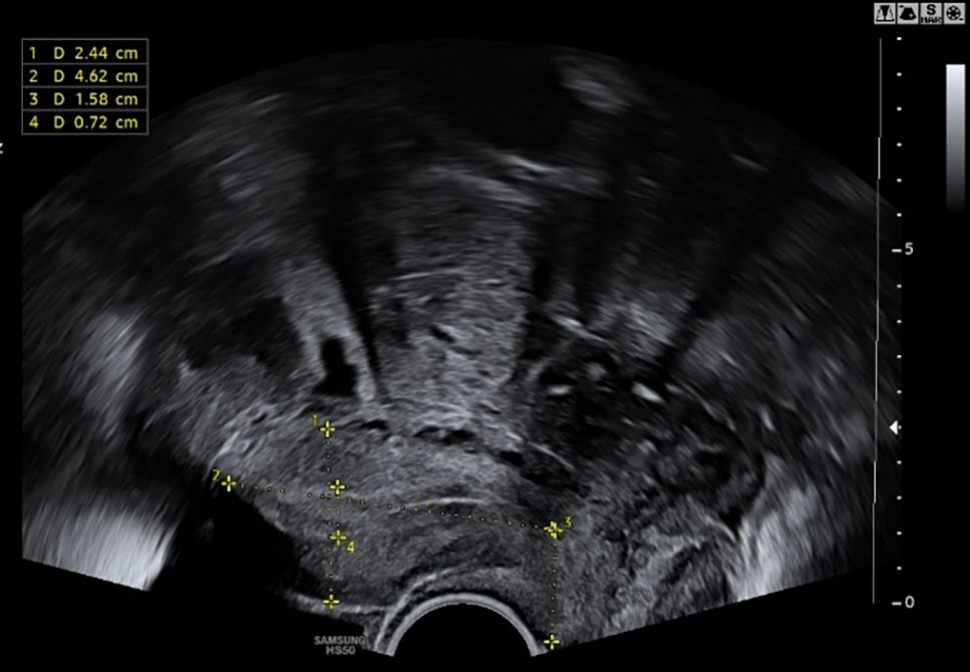
Unremarkable transvaginal sonogram of the uterus

**Fig. 5:**
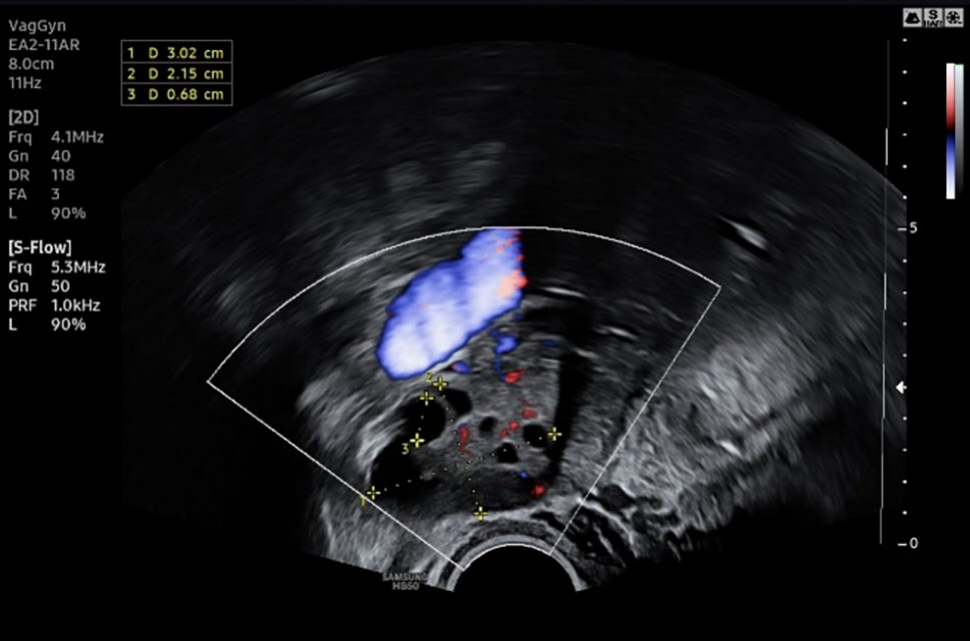
Unremarkable transvaginal sonogram of the right ovary

**Fig. 6:**
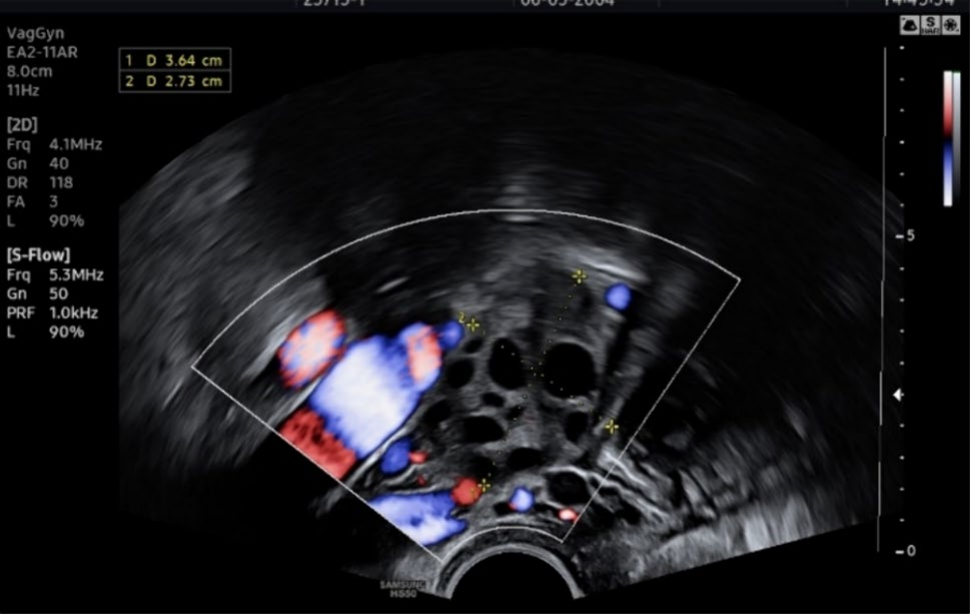
Unremarkable transvaginal sonogram of the left ovary

The 18-year-old patient presented with irregular vaginal bleeding at her gynecologist in July 2022. The clinical examination showed an exophytic tumor of the cervix consistent with a myoma in statu nascendi (see Figs. 1, 2, 3). Uterus and ovaries were normal in sonogram (see Figs. 4, 5, 6).

The tumor of the cervix was resected, followed by a diagnostic hysteroscopy and abrasion of the uterine cervix and cavity. Hysteroscopy showed normal findings of the cervix and uterus.

First histopathological results pointed to an embryonic rhabdomyosarcoma. Tumor size was 8.5 × 7 × 2.5 cm. TNM (tumor, nodes, metastasis) tumor stage was cT1 cN0 cM0 cR1. In microscopic evaluation, a polypoid, spindle-cell tumor mass consisting of myxoid parts, closely packed cells and a high rate of mitosis was seen. Tumor mass contained fetal cartilage.

Immunohistological staining showed positivity for CD 56, calponin and p53 (wildtype). Weak positivity for panCK was shown in less than 5% of tumor cells. Tumor cells were negative for CD34, S100, smooth muscle actin, desmin, Myo-D1 and caldesmon. Progesterone and estrogen receptors were not detected in tumor tissue. Tumor cells displayed up to 15 mitosis per high-power field. Ki-67 was 90%.

Due to the atypical immunohistological staining, samples were sent to a referral institute of pathology to obtain a second opinion.

Referral pathology confirmed a highly proliferative spindle cell neoplasia containing fetal cartilage. Additional immunohistological staining showed positivity for CD10. Mutational analysis revealed a DICER1 mutation in exon 24. Thus, a DICER1-associated highly malignant spindle-cell sarcoma with rapid tumor growth was diagnosed. According to the FNCLCC (Fédération Nationale des Centres de Lutte Contre le Cancer) grading system the tumor was categorized as grade 3.

Additionally, NTRK (**n**eurotrophic **t**ropomyosin or **t**yrosine **r**eceptor **k**inase genes (1–3)) testing was done, but revealed no anomalies.

Genetic testing in peripheral blood detected no germ line mutation in the DICER gene.

Tumor staging via CT (computed tomography) scan of the thorax and MRI (magnetic resonance imaging) of the abdomen and pelvis revealed no metastases or visible tumor rest.

In September 2022, the patient received counselling on fertility-preservation. In accordance with the patient`s preferences, cryopreservation of oocytes was realized.

This exceptional case of a highly malignant spindle-cell tumor of the uterine cervix in an 18-year-old patient was discussed in depth at the interdisciplinary tumor board. Despite the rarity of literature of similar cases, four cycles of chemotherapy consisting of doxorubicin and ifosfamide were recommended. Chemotherapy was applied from September to December 2022. After re-staging in December 2022 via CT scan and MRI, the abdomen and pelvis was unremarkable.

In January 2023, a control hysteroscopy, abrasion of the uterine cervix and cavity, as well as a biopsy of a minor lesion of the cervix revealed no evidence of tumor recurrence or residual disease. Additionally, no DICER mutation was found in the tissue, further supporting the absence of genetic abnormalities associated with the tumor. The patient was included in the German prospective registry for gynecological sarcoma (REGSA), the largest registry for gynecological sarcomas in Germany, Austria and Switzerland [2].

Repeated control hysteroscopy and abrasion in August 2023 showed the same, unremarkable results. CT scan and MRI the abdomen and pelvis in April and July 2023 revealed no sign of tumor recurrence.

Until now, November 2023, the patient is in remission and regular follow-ups including gynecological exam and sonography are performed.

## Discussion

Mutations in DICER1 (either somatic or germline) have been detected in a wide range of sarcomas [3]. A review of the literature shows that almost all gynecologic embryonal rhabdomyosarcomas reported (outside of the vagina) and 20% of adenosarcomas harbor DICER1 alterations [3]. Pathogenic germline variants in this gene cause a hereditary cancer predisposition syndrome—DICER 1 syndrome. Therefore, affected patients should be referred to genetic counselling [3]. In our patient with a somatic DICER1-associated sarcoma, germline mutations were not detected.

Rarely, sarcomas harbour a neurotropic tropomyosin receptor kinase (NTRK)-mutation. Given the responsiveness to selective NTRK inhibitors (e.g., larotrectinib), these ultra-rare cases of sarcomas harboring a NTRK-mutation should be identified [4]. To date, around 30 NTRK-rearranged mesenchymal tumors have been described in adult viscera, with a striking predominance in the uterine cervix [4]. However, NTRK testing in our patient revealed no anomalies.

Recent literature hints towards a distinct DICER-1 sarcoma entity characterized by specific mutational clusters [5, 6]. Characteristic pathological features, which are also present in our case, are subepithelial layer of malignant mesenchymal cells, areas of rhabdomyoblastic differentiation with positive staining with myogenin and myoD1, cellular/immature and occasionally malignant cartilage and areas of anaplasia [5, 6]. A simplified nomenclature such as “primary cervical sarcoma, Dicer-1 mutant” has been proposed [6].

So far, limited follow-up data suggest that DICER1-mutant tumors might be less aggressive than DICER1-wild type tumors with a 5-year overall survival rate of 78% and a progression-free survival rate of 58% [6, 7]. Main risk factors for relapse were IRS (Intergroup Rhabdomyosarcoma Studies) clinical group greater than I (i.e. tumor cannot completely be removed by surgery), tumor size greater than 5 cm, lymph nodal involvement, and non-embryonal histology [6, 7].

Due to the limited data in the literature on this specific entity and tumor site treatment was largely orientated on existing guidelines referring to soft tissue sarcomas. As the most important pillar of treatment complete surgical resection sparing fertility in this young patient was performed. Owing to the highly malignant nature and high proliferation rate of the tumor, we opted for adjuvant chemotherapy with four cycles of doxorubicin and ifosfamide. Prior to starting chemotherapy fertility-preservation measures consisting of cryoconservation of oocytes were realized. The German FertiPROTEKT network recommends fertility-protecting measures if (a) there is a good chance of survival (b) risk of permanent amenorrhea is above 20% (c) fertility-protecting measures are feasible and without major risk for the patient [8, 9]. Gonadotoxicity is usually measured by the risk of developing chemotherapy-induced amenorrhea (CIA). While temporary CIA after completion of chemotherapy can be observed in the majority of chemotherapy regimen the risk of persistent amenorrhea over the course of 6–12 months is classified into a high (> 80%), medium (40–60%) or low (< 20%) risk category. Permanent amenorrhea is not only closely linked to an unfulfilled desire for children, but also early development of menopause leading to an increased risk of osteoporosis, cardiovascular disease and psychosocial implications.

At an age of 18 years, a sterilizing effect of radiation would be reached at 16–18 Gy. Even a dosage of 2.5–5 Gy leads to a 60% risk of premature ovarian insufficiency between the age of 15–40 years. Only a dosage below 0.6 Gy seems to be associated with no additional risk of infertility in later life [8–10]. If radiation of the pelvis is indicated surgical transposition of the ovaries is an option to preserve ovarian function [9–12]. However, while radiation is widely used in sarcomas of soft tissue and bones, sensitivity to radiation in several gynecological sarcomas is low [13].

Gonadotoxicity of chemotherapy depends on the regimen used, total dosage and age of the patient. Whereas for example cyclophosphamide exhibits a highly gonadotoxic potential, anthracycline-based chemotherapy (e.g. doxorubicin) falls within the low-risk category (10–20% risk) when applied at a young age (< 30 years) accumulating to a risk of chemotherapy-induced amenorrhea of ~ 33% when applied between 31 and 35 years [13]. In the literature, data on gonadotoxic potential of ifosfamide is scare. However, analysis of a limited number of patients suggests a rather low gonadotoxic risk [14].

Based on the principle of obtaining maximum oncological safety while preserving fertility in this 18 year old patient with this rare entity we recommended chemotherapy rather than radiation with its far severe implications on the patient´s reproductive organs.

Until today, 1.5 years after initial diagnosis of Dicer-1-associated sarcoma of the cervix the patient is tumor-free and regular follow-ups including gynecological exam and sonography are performed.

## Conclusion

Decision-making in case of very rare histological entities with sparse recommendations in the literature poses a challenge to the treating physician. Especially rare tumors should be included in studies and registries such as the German REGSA registry of sarcomas to gain further insights into these entities.

Treatment strategies should consider oncological safety as well as options of preserving fertility.

Gonadotoxic potential of different strategies should be taken into consideration and discussed in detail with the affected patient.

## Data Availability

Data will be made available on request.
